# Modeling neurological diseases with induced pluripotent cells reprogrammed from immortalized lymphoblastoid cell lines

**DOI:** 10.1186/s13041-016-0267-6

**Published:** 2016-10-03

**Authors:** Koki Fujimori, Toshiki Tezuka, Hiroyuki Ishiura, Jun Mitsui, Koichiro Doi, Jun Yoshimura, Hirobumi Tada, Takuya Matsumoto, Miho Isoda, Ryota Hashimoto, Nubutaka Hattori, Takuya Takahashi, Shinichi Morishita, Shoji Tsuji, Wado Akamatsu, Hideyuki Okano

**Affiliations:** 1 Department of Physiology, Keio University, School of Medicine, Shinjuku-ku, Tokyo, 160-8582 Japan; 2 Department of Neurology, Graduate School of Medicine, The University of Tokyo, Bunkyo-ku, Tokyo, 113-8655 Japan; 3 Department of Computational Biology and Medical Sciences, Graduate School of Frontier Sciences, The University of Tokyo, Kashiwa, 277-0882 Japan; 4 Department of Physiology, Yokohama City University Graduate School of Medicine, Kanazawa-ku, Kanagawa 236-0027 Japan; 5 Department of Integrative Aging Neuroscience, Section of Neuroendocrinology, National Center for Geriatrics and Gerontology, Obu, Aichi 474-8511 Japan; 6 Institute for Innovation, Ajinomoto Co., Inc., Kawasaki-ku, Kanagawa 210-8681 Japan; 7 Molecular Research Center for Children’s Mental Development, United Graduate School of Child Development, Osaka University, Suita-shi, Osaka 565-0871 Japan; 8 Department of Psychiatry, Osaka University Graduate School of Medicine, Suita-shi, Osaka 565-0871 Japan; 9 Department of Neurology, Juntendo University, School of Medicine, Bunkyo-ku, Tokyo, 113-8431 Japan; 10 Medical Genome Center, The University of Tokyo Hospital, Bunkyo-ku, Tokyo, 113-8655 Japan; 11 Center for Genomic and Regenerative Medicine, Juntendo University, School of Medicine, Bunkyo-ku, Tokyo, 113-8431 Japan

**Keywords:** Lymphoblastoid B-cell line, Disease modeling, Neurological disorder, Induced pluripotent stem cells, Genomic mutation in reprogramming process

## Abstract

Patient-specific induced pluripotent stem cells (iPSCs) facilitate understanding of the etiology of diseases, discovery of new drugs and development of novel therapeutic interventions. A frequently used starting source of cells for generating iPSCs has been dermal fibroblasts (DFs) isolated from skin biopsies. However, there are also numerous repositories containing lymphoblastoid B-cell lines (LCLs) generated from a variety of patients. To date, this rich bioresource of LCLs has been underused for generating iPSCs, and its use would greatly expand the range of targeted diseases that could be studied by using patient-specific iPSCs. However, it remains unclear whether patient’s LCL-derived iPSCs (LiPSCs) can function as a disease model. Therefore, we generated Parkinson’s disease patient-specific LiPSCs and evaluated their utility as tools for modeling neurological diseases. We established iPSCs from two LCL clones, which were derived from a healthy donor and a patient carrying *PARK2* mutations, by using existing non-integrating episomal protocols. Whole genome sequencing (WGS) and comparative genomic hybridization (CGH) analyses showed that the appearance of somatic variations in the genomes of the iPSCs did not vary substantially according to the original cell types (LCLs, T-cells and fibroblasts). Furthermore, LiPSCs could be differentiated into functional neurons by using the direct neurosphere conversion method (dNS method), and they showed several Parkinson’s disease phenotypes that were similar to those of DF-iPSCs. These data indicate that the global LCL repositories can be used as a resource for generating iPSCs and disease models. Thus, LCLs are the powerful tools for generating iPSCs and modeling neurological diseases.

**Electronic supplementary material:**

The online version of this article (doi:10.1186/s13041-016-0267-6) contains supplementary material, which is available to authorized users.

## Introduction

Modeling with induced pluripotent cells (iPSCs) is highly useful in research of neural diseases [1–4] because it is difficult to obtain patient-derived cells from the central nervous system that recapitulates the disease pathology. Dermal fibroblasts (DFs) have widely been used as a source of patient-specific iPSCs because they were used to derive the first human iPSCs [5]. However, the isolation of DF cell lines requires invasive skin biopsies from patients. Recently, we have reported that T-cell derived iPSCs (TiPSCs) and fibroblast-derived iPSCs can be used to model neural diseases by using robust neural induction protocols [6]. TiPSCs can be established from a small amount of peripheral blood, such that patients can provide samples through a minimally invasive procedure [7]. Although peripheral blood cells, including T-cells, appear to be ideal sources for generating iPSCs, cloned and frozen T-cells are unstable, owing to their variable reprogramming efficiency depending on the donors and conditions of each sample (e.g., culture medium or number of passages) [8–10]. In particular, efficient wide-scale parallel reprogramming of samples requires a stable source of cells to achieve high-throughput processing and to model polygenic or sporadic diseases. Lymphoblastoid B-cell lines (LCLs) are stable peripheral B-cell lines that are transformed by infection with Epstein-Barr virus (EBV). LCLs are easy to maintain, and various types of well-characterized LCL clones established from patients are already available in worldwide repositories and are usually linked to patient clinical history, long-term genotype and phenotype data, and molecular/functional studies of various diseases [11, 12]. LCL banks have been of great importance in providing reference material for rare genetic diseases, as well as in managing large amounts of DNA for the genetic analysis of complex conditions in population- and family-based disease collections [13, 14]. A number of major facilities currently establish and manage extensive collections of cell lines (https://catalog.coriell.org/; http://ja.brc.riken.jp/; https://www.eagle-i.net/; www.ecacc.org.uk; www.alspac.bris.ac.uk; www.lgcpromochem-atcc.com; www.rutgers.edu) for the international research community.

To date, five groups have reported the successful reprogramming of LCLs from healthy donors and patients into iPSCs [15–19]. However, it is unclear whether LCL-derived iPSCs (LiPSCs) can be used for disease-specific analyses. Although immortalization of B-cells by EBV infection [20] may be a problematic procedure in terms of the cellular characteristics of LiPSCs, little is known about the effects of EBV infection on the properties of LiPSCs or even LCLs. Although EBV transformation commonly maintains the genome composition of the original cells [21], long-term LCL culture may cause several aberrations, including genomic aberrations such as chromosomal aneuploidy, down-regulation of p16/Rb, mutation of the p53 gene, modulation of apoptosis and sensitivity to various chemical agents [22]. Therefore, detailed analyses of the genomic structure and cellular properties of LCLs and LiPSCs are necessary because unexpected genomic mutations would confound the disease-specific phenotypes in disease models generated with iPSCs. Additionally, some genomic aberrations and/or epigenetic memories derived from the source cells can affect the differentiation ability of iPSCs [23–25].

Although neurological disease models generated with LiPSCs would accelerate the progress of patient-specific iPSC studies, these concerns regarding the effects of EBV must be elucidated. Here, we established LiPSCs from both healthy donor and patient with the mutation of *PARK2* (*parkin*) known as one of the causative genes for familial Parkinson’s disease,) to evaluate the characteristics of LiPSCs, including whole genomic sequencing and to confirm the utility of LiPSCs as tools for modeling neurological diseases.

## Results

### Establishment and characterization of iPSCs derived from LCLs

**Fig. 1:**
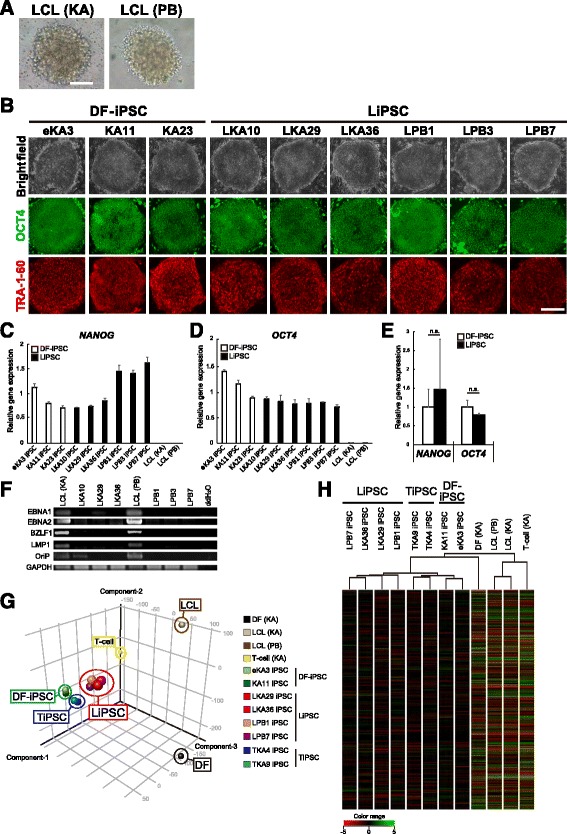
Establishment and characterization of LCL-derived iPSCs. **a** Representative image of an LCL culture. Scale bar = 100 μm. **b** iPSCs derived from healthy control LCLs (LKA10, LKA29 and LKA36), isogenic dermal fibroblasts (DFs) (eKA3, KA11, and KA23) and LCLs from a PARK2 patient (LPB1, LPB3 and LPB7) were immunopositive for the pluripotent markers OCT4 (green) and TRA-1-60 (red). Scale bar = 200 μm. **c**–**e** The expression levels of pluripotent markers *NANOG* and *OCT4* in LiPSCs (LKA10, LKA29 LKA36, LPB1, LPB3 and LPB7), DF-iPSCs (eKA3, KA11 and KA23) and LCLs (LCL(KA) and LCL(PB)) were assessed by quantitative reverse-transcription PCR (qPCR). The values from the previously established DF-iPSCs (201B7, a previously established human iPSC clone [5]) were set to 1.0 (*n* = 3 independent experiments; means ± SEM; n.s., not significant; Student’s *t*-test). **f** The expression levels of EBV-related genes (*EBNA-1*, *EBNA-2*, *BZLF-1*, *LMP-1* and *OriP*) were analyzed by a PCR analysis of the genomic DNA obtained from parental LCLs and LCL-derived iPSCs. *GAPDH* was used a loading control. **g** Comparison of the global gene expression profiles of DF-iPSCs (eKA3 and KA11), LiPSCs (LKA29, LKA36, LPB1 and LPB7), TiPSCs (TKA4 and TKA9) [6], and the original cells (DF(KA), LCL(KA), LCL(PB) and T-cell(KA)). Principal component analysis of the gene expression data. Black: DF, Brown: LCLs, Yellow: T-cell, Green: DF-iPSCs, Red: LiPSCs, Blue: TiPSCs. **h** Hierarchical clustering analysis of the global gene expression profiles. The data discussed in this publication have been deposited in the NCBI Gene Expression Omnibus (GEO, http://www.ncbi.nlm.nih.gov/geo/) database and are accessible through GEO Series accession numbers GSE76832 [6] and GSE82159

Previous reports have shown that reprogramming LCLs to iPSCs by using existing non-integrating episomal protocols provided no identifiable iPSC clones, even after 35–40 days [17, 18]. Recently, R. Barrett and colleagues have developed a new episomal (OriP/EBNA1) plasmid reprogramming method for LCLs, which reduces the time required to generate LiPSCs and enhances the efficiency of reprograming LCLs into iPSCs [15]. We modified this method for on-feeder cultures and applied it to LCLs from two donors: a healthy person, “KA” and a patient with autosomal recessive juvenile Parkinson’s disease (PARK2), “PB” (Fig. 1a). LiPSCs were generated with a similar efficiency to that of a previously described method [15], and the reprogramming efficiencies of LKA and LPB were 0.002 and 0.0015 %, respectively. After optimizing the protocol for generating iPSCs from LCLs, we established several LiPSC clones, including three healthy donor-derived LiPSC lines, LKA10, LKA29, and LKA36 and four PARK2 patient-derived LiPSC lines, LPB1, LPB3, LPB7 and LPB8, which had a normal karyotype (Additional file 1: Figure S1A). In addition, we used four DF-iPSC lines derived from the same healthy donor, eKA3, eKA4, KA11 and KA23, as the control reference lines [6] in this study.

Representative morphologies of LiPSC colonies are shown in Fig. 1b. All LiPSC lines exhibited typical PSC characteristics, including tightly packed colonies, a high cell nuclear-cytoplasmic ratio, and the production of the surface and nuclear pluripotency proteins, TRA1-60 and OCT4 and were indistinguishable from the DF-iPSC lines (Fig. 1b). LiPSCs also expressed endogenous pluripotency genes at a similar level to DF-iPSCs (Figs. 1c–e). These data indicated that LiPSCs and DF-iPSCs were indistinguishable in terms of morphology and the expression of pluripotent markers at both the protein and mRNA levels (Fig. 1b–e). In addition, we confirmed whether LiPSCs have differentiation potentials into three-germ layers by in vitro differentiation analysis via EB (Additional file 1: Figure S1B and C). Thus, all LiPSC clones had a pluripotency. Because EBNA-1 has been reported to be required for the establishment of persistent EBV infection and survival of host B-cells [26], we next examined the expression of *EBNA1* and additional EBV-related genes (*EBNA-2*, *BZLF-1*, *LMP-1* and *OriP*) in LiPSCs. A PCR analysis of genomic DNA showed that all the EBV-related latency elements were eventually eliminated from the established LiPSCs, suggesting the loss of EBV-associated elements as a result of the reprogramming process (Fig. 1f).

The global gene expression profiles in iPSCs at passages below 20 were evaluated with a microarray analysis to explore the detailed differences between LiPSCs, TiPSCs and DF-iPSCs resulting from the origin of the iPSCs. With the exception of the genes that were expressed at low levels in all samples, the data were normalized and subjected to principal component analysis (PCA) (Fig. 1g) and hierarchical clustering (Fig. 1h). Although the LiPSCs, TiPSCs and DF-iPSCs were relatively close to each other in the PCA analysis (Fig. 1g), hierarchical clustering placed the LiPSCs, TiPSCs and DF-iPSCs into different groups (Fig. 1h). These data also suggested that the original cell type influences the properties of hiPSCs.

### Immortalization by EBV does not affect the number of *de novo* mutations and structural variations in LiPSCs

**Fig. 2:**
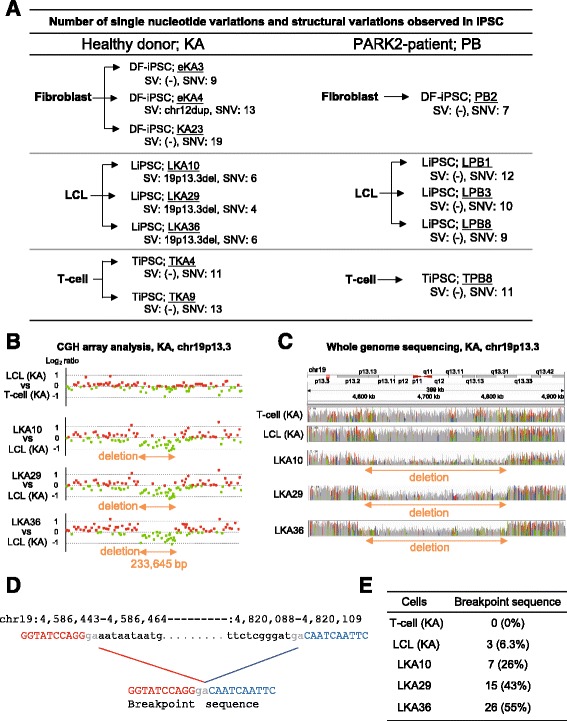
*De novo* mutations and structural variations caused by the reprogramming process. **a** Summary of the number of somatic mutations. SVs were detected by an aCGH analysis. Candidate SNVs were identified by whole genome analysis and confirmed by a direct nucleotide sequence analysis. Only the nonsynonymous variants in protein coding regions outside the immunoglobulin or T-cell receptor gene regions are shown. **b** A recurrent structural variation in the short arm of chromosome 19 in healthy donor KA detected by CGH analysis is shown. The deletion was detected in all the LiPSC clones examined, but was not detected in the LCLs. **c** The coverage data for each sample (LKA10, LKA29 and LKA36), as shown by the Integrative Genomic Viewer [44]. In the LiPSC clones, decreased coverages were observed and corresponded to the deletions detected by the CGH analysis. **d** The breakpoint sequences were identified in the short reads obtained from the LCLs. Identical breakpoint sequences were also identified in the short reads obtained from LKA10, LKA29 and LKA36. There is a 2 bp microhomology at the breakpoint. **e** The short read sequences indicating the breakpoint were identified in only 6.3 % of the total reads spanning this region in LCLs. Short read sequences indicating the same breakpoint were identified in all the LiPSC clones. These short read sequences were observed in 26, 43 and 55 % of the total reads obtained from LKA10, LKA29 and LKA36 clones, respectively

We performed array-based comparative genomic hybridization (aCGH) and whole genome sequence (WGS) analyses to examine the somatic structural variations (SVs) and single nucleotide variations (SNVs) in LiPSCs (Fig. 2a). A comparison of the genomes of the LiPSC clones and LCLs revealed a deletion (233,645 bp) at 19p13.3 in all the LiPSC clones examined from the healthy donor, KA (Fig. 2b–d). Although the number of reads was limited (approximately 6 % of the total reads), the presence of the reads spanning the breakpoint was confirmed not only in the LiPSC clones but also in the LCLs (Fig. 2e), thus strongly suggesting that the deletions in the LiPSC clones were already present in a subpopulation of their original cells, LCLs and were not detected by the aCGH analysis.

We then searched for the appearance of SNVs by comparing the LiPSC clones with LCLs by using the WGS analysis. In this analysis, we focused on non-synonymous SNVs in coding regions, and all the variations were further validated by direct nucleotide sequence analysis. The analysis revealed the appearance of 4–6 non-synonymous variations in the LiPSCs clones derived from KA. The analysis revealed the appearance of 9–12 non-synonymous SNVs in the PB-LiPSC clones compared with the original cell source, LCLs (Fig. 2a).

We identified a somatic mutation in SLC26A5 (rs758296903) in all the LiPSC clones from KA (LKA10, LKA29 and LKA36) compared with their original cells. A detailed examination of the short reads revealed that 8.6 % of the reads from the LCLs carried the mutation, thus indicating that the SNV was already present in a subpopulation of the original cells. Other variations observed in the TiPSCs and DF-iPSCs compared with T-cells and DFs, respectively, are shown in Fig. 2a and Additional file 1: Figure S1. These data indicate that the reprogramming and/or immortalization processes might cause some somatic mutations. However, the total number of somatic mutations observed in the genomes of iPSCs compared with their corresponding original cells did not vary among the cell sources of origin (LCLs, T-cells and fibroblasts: Fig. 2a), thus suggesting that LiPSCs may also have the same properties and functions as hiPSCs, similarly to TiPSCs and DF-iPSCs.

### Differentiation of neural cells from LiPSCs through a direct neurosphere conversion method

**Fig. 3:**
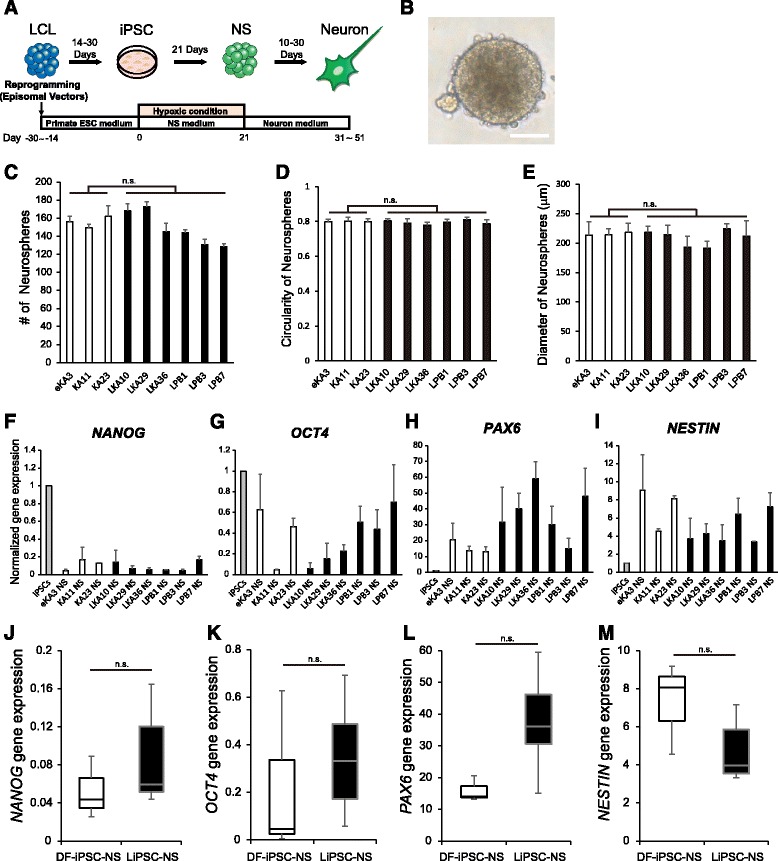
Comparison of LiPSC- and DF-iPSC-derived neurospheres. **a** Overview of the culture protocols used in this experiment. **b** Representative image of floating NSs derived from LiPSCs. Scale bar = 100 μm **c** NS formation assay using LiPSCs and DF-iPSCs. NSs with a diameter greater than 50 μm were counted (*n* = 6 independent experiments; means ± SEM; n.s., not significant; Student’s *t*-test). **d**–**e** Morphological analysis of NSs derived from LiPSCs and DF-iPSCs. The circularity and averaged diameter of NSs were quantified (*n* = 6 independent experiments; means ± SEM; n.s., not significant; Student’s *t*-test). **f**–**i** Expression levels of pluripotent markers (*NANOG* and *OCT4*) and neural stem markers (*PAX6* and *NESTIN*) in NSs derived from DF-iPSCs (eKA3, KA11 and KA23) and LiPSCs (LKA10, LKA29, LKA36, LPB1, LPB3 and LPB7). The expression levels were normalized by setting the values from 201B7 [5] to 1.0 (*n* = 3 independent experiments; means ± SEM). **j**–**m** Box-and-whisker plots showing the mRNA transcript levels in NSs derived from DF-iPSCs and LiPSCs. (*n* = 3 independent experiments; means ± SEM; n.s., not significant; Student’s *t*-test)

In our previous reports, we have shown that TiPSCs are poorly differentiated into the ectodermal lineage, and it is difficult to induce TiPSCs to differentiate into neuronal cells via EB formation by using spontaneous neuronal differentiation protocols. To overcome this limitation, we developed a neurosphere (NS)-based differentiation method (direct NS conversion method: dNS method). TiPSCs were differentiated into neural cells with similar efficiency as DF-iPSCs with the dNS method [6]. We used the dNS method to differentiate the LiPSCs into neural cells (Fig. 3a) because both TiPSCs and LiPSCs were derived from peripheral blood cells and supposedly have similar differentiation propensities. With the dNS method, LiPSCs efficiently formed similar number of NSs (Fig. 3b) compared with DF-iPSCs (Fig. 3c). Morphological analysis of NSs focusing on their circularity (Fig. 3d) and diameter (Fig. 3e) also demonstrated that there were no significant differences between LiPSC- and DF-iPSC-NSs (Figs. 3d and e). We quantified the expression of pluripotent markers, *NANOG* and *OCT4* and neural markers, *PAX6* and *NESTIN*, in LiPSC-derived NSs by qPCR analysis. Although the expression of *NANOG* in LiPSC-derived NSs was lower in all clones and DF-iPSC-derived NSs (Fig. 3d), *OCT4* expression was maintained in some LiPSC-derived NS clones and DF-iPSC-derived NS clones (Fig. 3e). However, these differences in *NANOG* (Fig. 3h) and *OCT4* expression (Fig. 3i) were not statistically significant among the cell types of origin. The expression of neural stem cell markers did not differ significantly among the various NSs we tested, regardless of the original cell type and donor (Fig. 3f, g, j and k). These results indicate that LiPSCs can differentiate into NSs via the dNS method as efficiently as DF-iPSCs.

### LiPSCs can be differentiated into various types of functional neurons to a similar level as DF-iPSCs by using the dNS method

**Fig. 4:**
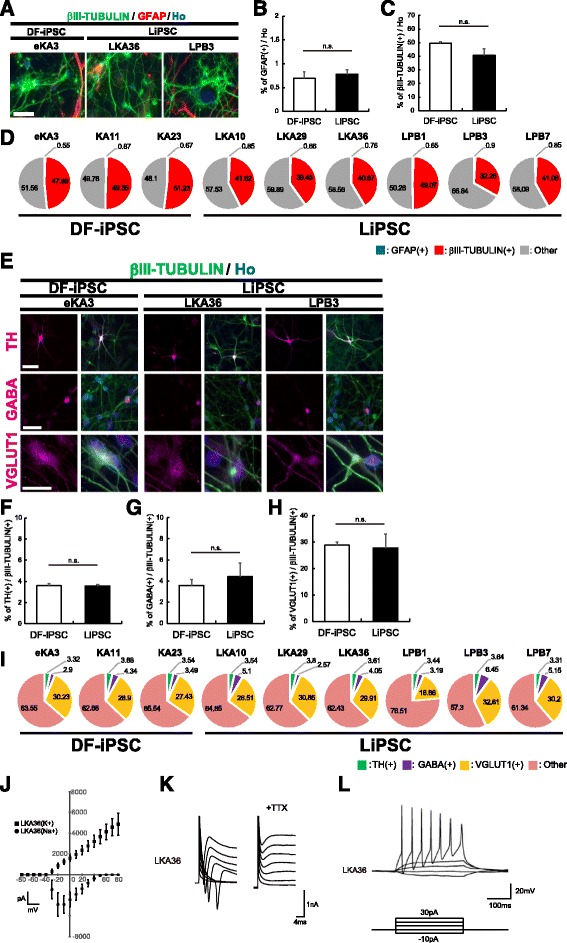
Generation of various types of functional neurons from LiPSCs with the dNS method. **a** Representative images of DF-iPSC- and LiPSC-derived neurons and glial cells stained with antibodies against the indicated markers. Scale bars = 50 μm. **b**–**c** Ratio of cell types differentiated from DF-iPSCs and LiPSCs (*n* = 3 independent experiments; means ± SEM; n.s., not significant; Student’s *t*-test). **d** Summary of cell types produced from each hiPSC line (*n* = 3). **e** Representative images of DF-iPSC- and LiPSC-derived neuronal subtypes stained with antibodies against the indicated markers. Scale bars = 50 μm. **f**–**h** Ratio of neuronal subtypes differentiated from DF-iPSCs and LiPSCs (*n* = 3 independent experiments; means ± SEM; n.s., not significant; Student’s *t*-test). **i** Summary of neuronal subtypes produced from each hiPSC line (*n* = 3). **j** Current-voltage plot of the sodium and potassium currents of neurons derived from LiPSCs (*n* = 6 independent experiments; means ± SEM). **k** Voltage-dependent sodium and potassium currents in neurons derived from LiPSCs. **l** Representative traces of the membrane potential of LiPSC-derived neurons in response to step depolarization by a current injection

We next examined whether LiPSC-derived NSs could be induced to differentiate into neural cells as efficiently as DF-iPSCs. LiPSC-derived NSs were dissociated into single cells and cultured for neural differentiation. Thirteen days later, the differentiated cells were immunostained with antibodies against a neural marker, βIII-TUBULIN and an astrocyte marker, glial fibrillary acid protein (GFAP) (Fig. 4a). LiPSC-derived NSs differentiated into neurons and astrocytes at a similar ratio as DF-iPSCs, indicating that LiPSC-derived NSs had similar propensities to differentiate into neurons and astrocytes as the DF-iPSC-derived NSs (Fig. 4b–d). The immunocytochemical analysis revealed that these βIII-TUBULIN-positive neurons included tyrosine hydroxylase (TH)-positive dopaminergic neurons (Fig. 4e and f), gamma-aminobutyric acid (GABA)-positive GABAergic neurons (Fig. 4e and g), and vesicular glutamate transporter 1-positive glutamatergic neurons (Fig. 4e and h). Figs. 4d and i show a summary of the data from these differentiated cell types and neuronal subtypes, suggesting that LiPSCs differentiated into various types of cells as efficiently as DF-iPSCs, regardless of the differences among donors.

We recorded voltage-sensitive currents in 60-day-old LiPSC-derived neurons to confirm their electrophysiological properties. LiPSC-derived NSs were infected with a lentivirus expressing a human *Synapsin I* promoter-driven GFP (CSIV–hSynI-GFP-IRES2-NeoR) soon after single dissociated NS cells were plated [27]. After neuronal maturation, voltage-dependent Na^+^ and K^+^ currents and TTX-sensitive voltage-gated membrane currents were detected (Fig. 4j and k), and action potentials were elicited in the majority of the LiPSC-derived neuronal cells analyzed by depolarizing the membrane in current clamp mode (Fig. 4l). These results suggested that LiPSCs can be differentiated into functional neurons through the dNS method.

### Neurons derived from PARK2-LiPSCs exhibited impaired mitochondrial activity and phenotypes

**Fig. 5:**
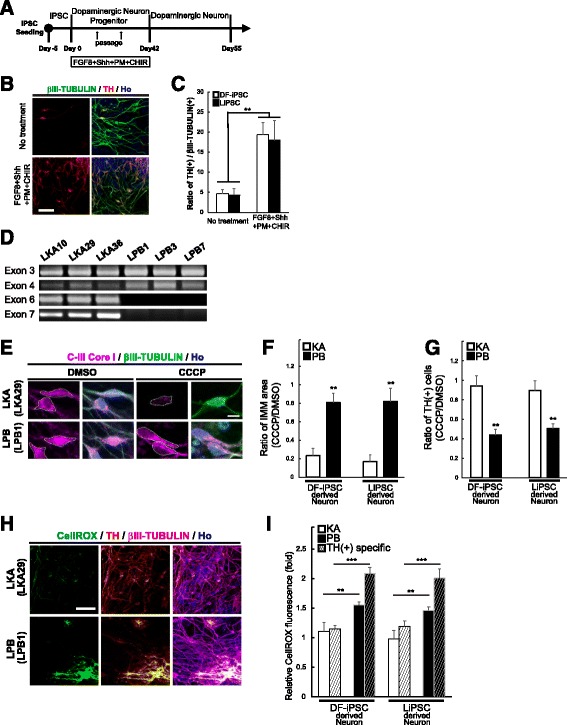
Reproduction of Parkinson’s disease phenotypes using neurons derived from PARK2-LiPSCs. **a** An overview of the culture protocol used in this experiment. **b** Immunostaining of LiPSC (LPB10)-derived dopaminergic neurons with antibodies raised against the indicated markers. Scale bars = 100 μm. **c** Analysis of dopaminergic neuron differentiation by quantifying βIII-TUBULIN and TH double-positive neurons at Day 55 (*n* = 3 independent experiments; means ± SEM; **p < 0.01; Student’s *t*-test). **d** The deletion of exons 6 and 7 was confirmed in clones LPB1, LPB3 and LPB7. **e** Double labeling for the IMM marker CIII-Core I and βIII-TUBULIN in DMSO- or CCCP-treated DF-iPSC- and LiPSC-derived neurons from a healthy donor or a PARK2 patient. Scale bar = 50 μm. **f** The ratio of the IMM area was determined by quantifying the CCCP/DMSO ratio of CIII-Core I staining in the βIII-TUBULIN-positive cells (*n* = 3 independent experiments; means ± SEM; **p < 0.01; Student’s *t*-test). **g** The CCCP treatment significantly decreased the ratio of TH-positive neurons in the PARK2 patient-derived neurons compared with the control (*n* = 3 independent experiments; means ± SEM; **p < 0.01; Student’s *t*-test). **h**, **i** Analysis of oxidative stress using the CellROX® Green Reagent. The PARK2 neurons showed increased CellROX® fluorescence compared with the control neurons (*n* = 3 independent experiments; means ± SEM; **p < 0.01, ***p < 0.001; Student’s *t*-test). Scale bars = 500 μm

We differentiated LiPSCs derived from a patient with *parkin* mutations, a familial form of Parkinson’s disease, PARK2, into neurons to determine whether hiPSCs established from LCLs could be used as a model of neurological disease. A consistent neurochemical abnormality found in Parkinson’s disease is the degeneration of dopaminergic neurons in the substantia nigra. Therefore, we exposed single dissociated hiPSCs to FGF8, sonic hedgehog (Shh), purmorphamine (PMA) and CHIR99021 during the NS formation, as described in our previous study [6], to generate midbrain dopaminergic neuron (mDAN)-enriched culture. After neuronal maturation for 13 days in culture, immunostaining of the neural epithelial cells demonstrated that nearly 20 % of the βIII-TUBULIN-positive cells expressed the dopaminergic neuron marker TH, with a similar differentiation ratio between the DF-iPSCs and LiPSCs (Fig. 5b and c). These data indicate that the LiPSCs and DF-iPSCs were able to differentiate into mDANs with similar efficiencies (Fig. 5a–c).

A PCR analysis of the genomic DNA confirmed that PARK2 patient-derived LiPSCs (LPBs; LPB1, LPB3 and LPB7) contained a homozygous deletion in exons 6 and 7 of the *parkin* gene (Fig. 5d), which encodes a component of an E3 ubiquitin ligase involved in mitochondrial homeostasis [28]. We have previously reported that hiPSCs established from DFs of a PARK2 patient exhibited abnormal turnover of damaged mitochondria [29] and increased production of reactive oxygen species (ROS) in their neurons. Therefore, we treated the LiPSC-derived neurons with carbonyl cyanide m-chlorophenyl hydrazone (CCCP), which triggers the loss of mitochondrial membrane potential and results in the removal of damaged mitochondria. We visualized the area of the inner mitochondrial membrane (IMM) by using an antibody against the IMM marker Complex-III Core I (C-III Core I) to determine the extent to which the damaged mitochondria were eliminated after CCCP treatment. Compared with the untreated samples, the CCCP-treated samples elicited a dramatic decrease in the IMM area in the control neurons (LKA clones), but not in the PARK2 neurons (LPB clones) (Fig. 5e and f). Using the same CCCP-treated neuronal samples, we performed immunostaining with an anti-TH antibody to quantify the ratio of TH-positive dopaminergic neurons. In both the DF-iPSC- and LiPSC-derived neurons, we observed significant decreases in the numbers of TH-positive neurons in PARK2-derived cells (LPB clones) after the CCCP treatment, indicating that dopaminergic neurons derived from PARK2-iPSCs were more vulnerable to mitochondrial stress than control-iPSCs (Fig. 5g).

Finally, we evaluated ROS production in the neurons derived from the control- and PARK2-LiPSCs by using the CellROX® Green Reagent, which is weakly fluorescent in a reduced state and exhibits bright green photostable fluorescence after oxidation by ROS, with absorption/emission maxima of ~ 485/520 nm. The reactive CellROX fluorescence was significantly increased in the PARK2 neurons, and this phenotype prominently appeared in the TH-positive neurons, indicating that ROS production was increased in the PARK2 neurons, particularly the mDANs (Fig. 5h and i). These phenotypes were observed in both DF-iPSC- and LiPSC-derived neurons (Figs. 5e–i). These results strongly suggest that the hiPSCs derived from patient LCLs can be used as models of neurological diseases.

## Discussion

Patient-specific iPSC technology makes it possible to recapitulate disease phenotypes in vitro, thus significantly facilitating the elucidation of disease processes and the development of therapeutic drugs. However, invasive skin biopsies are generally performed to obtain a patient’s original cells, DFs and generate iPSCs. Several groups have succeeded in establishing iPSCs from peripheral blood cells, T-cells and LCLs, thus extending the use of iPSC technologies [15–19, 30, 31]. In particular, our previous study has demonstrated the utility of TiPSCs as tools for modeling diseases. However, it had been unclear whether LiPSCs can function as a disease model. Because LCLs are stable cell lines that are easy to culture, they are expected to be the ideal resource for efficient wide-scale parallel reprogramming to achieve high-throughput processing for modeling polygenic or sporadic diseases. Moreover, various types of LCL clones established from patients are already available and stored in global repositories [12]. Therefore, it would be very important to obtain experimental proof of the utility of LiPSCs as disease models to increase the number of cases, perform disease-specific analyses based on statistical analysis, and expand the target-disease areas for iPSC technology.

In addition to this study, several groups have established iPSCs from peripheral blood cells and have investigated their characteristics as iPSCs [6, 7, 15–19, 30, 31]. However, little is known about the effects of the differences among original cells on genomic mutations in the derived iPSCs. In particular, LiPSCs are highly influenced by the original cells because LCLs are generated through an EBV infection-mediated immortalization process. This study clearly showed that the differences among LCLs compared with the original cells had no substantial effects on the number of somatic single nucleotide variations and structural variations in the established iPSCs compared with those obtained from T-cells or DFs (Fig. 2). These data broaden the applications of LiPSCs, and strongly support the utility of LCLs as original cells for generating iPSCs. Moreover, our results indicated that the reprogramming processes can cause some somatic mutations, regardless of the cell type of origin (Fig. 2 and Additional file 2: Figure S2). Although some of these mutations have recently been described [32], further studies are needed to clarify their effects.

In recent studies of human clinical genetics, progress has been made in the identification of the genes responsible for neurological disorders [33, 34] and the genes increasing the onset risk of neuropsychiatric disorders [35]. However, there are many diseases for which the causative genes and genetic risk factors have not yet been identified. Recent whole exome sequencing studies have revealed that these sporadic cases are related to many variants, indicating the cumulative genetic mutations that trigger the onset of the disease. It is important to establish hiPSCs from a sufficient number of patients and characterize multiple clones for statistical analyses to elucidate the pathogenic mechanisms of these diseases. Well-characterized LCLs are already available in global repositories and are linked to patient clinical history, long-term genotype and phenotype data, and molecular/functional studies. Then, the clarification of phenotypes using hiPSC disease model could elucidate the pathological mechanism of such sporadic diseases, and provide a path to the identification of their related genes. Therefore, in addition to T-cells, LCLs are a suitable source for generating hiPSCs to model sporadic or common diseases.

## Methods

### Human iPSC generation from LCLs

Peripheral blood mononuclear cells (PBMCs) were obtained from a healthy donor, “KA” and a patient with autosomal recessive juvenile Parkinson’s disease (PARK2), “PB”, by centrifuging heparinized blood over a Ficoll-Paque PREMIUM (GE Healthcare, Chicago, USA) gradient. KA- or PB-derived PBMCs were immortalized by an EBV infection according to the protocol of SRL Medisearch Incorporation and were transformed into LCLs. LCLs were cultured in RPMI 1640 (Gibco, Massachusetts, USA) supplemented with 10 % fetal bovine serum (FBS) at 37 °C and 5 % CO_2_ in a humidified incubator. After several passages, the LCLs were electroporated with the Neon™ Transfection System 100 μL Kit (MPK10096; Thermo Fisher Scientific, Massachusetts, USA) using 1.0 μg of each episomal plasmid (Addgene, Cambridge, USA) expressing 6 factors: *OCT4, SOX2*, *KLF4*, *l-MYC*, *LIN28* and *p53* shRNAs (pCXLE-hOCT3/4-shp53, pCXLE-hSK and pCXLE-hUL), according to the manufacturer’s instructions. The transfected LCLs were rapidly transferred to a 6-well plate at a density of 2.0 × 10^6^ cells/well and incubated for 24 h. At 24 h after electroporation, the medium was replaced with hiPSC medium. After an additional 24 h, the cells were passaged to a 100 mm dish containing mitomycin C-inactivated mouse SNL feeder cells at a density of 5.0 × 10^4^–5.0 × 10^5^ cells/dish and cultured in hiPSC medium, which was changed every other day until colonies were picked. The generated hiPSCs were maintained on mitomycin C-inactivated mouse SNL feeder cells in hiPSC medium. All hiPSC lines analyzed in this study were between passage 7 and 20.

All human primary cells were used after appropriate written informed consent was given to the commercial providers. All experimental procedures for biopsy and reprogramming were approved by the Ethics Committee of the Keio University School of Medicine (No. 20080016).

### Neural differentiation in vitro

The dNS method, which we have previously reported, was used for the neural differentiation of hiPSCs [6]. For neural induction, hiPSCs were dissociated into single cells by incubation with TrypLE^TM^ Select (Life Technologies, Massachusetts, USA) for 5 min and pipetting. The cells were cultured at a density of 10 cells/μL in a T25 flask (Nunclon, Massachusetts, USA) in MHM supplemented with B27 (Gibco, Massachusetts, USA), 20 ng/mL FGF-2 (Wako, Osaka, Japan), 10 μM Y-27632 (Wako, Osaka, Japan) and 10 ng/mL hLIF (Nacalai Tesque, Kyoto, Japan) in 4 % oxygen for 7 days. NSs were repeatedly passaged by dissociation into single cells, and then cultured at a density of 50 cells/μL in the same manner as the primary sphere formation. NSs were used at passage 3 for analysis. For terminal differentiation, the dissociated NSs were plated on PO- (Sigma-Aldrich, Missouri, USA) and fibronectin- (Sigma-Aldrich, Missouri, USA) coated coverslips and cultured in MHM containing B27 (Gibco, Massachusetts, USA), 10 ng/mL brain-derived neurotrophic factor (BDNF; R&D Systems, Minnesota, USA), 10 ng/mL glial cell-differentiated neurotrophic factor (GDNF; R&D Systems, Minnesota, USA), 200 μM ascorbic acid (Sigma-Aldrich, Missouri, USA) and 1 mM dibutyryl-cAMP (Sigma-Aldrich, Missouri, USA) for 10–60 days.

### Immunocytochemical analysis of hiPSCs and neurons derived from hiPSCs

Cells were fixed in phosphate-buffered saline (PBS) containing 4 % paraformaldehyde (PFA) for 30 min at room temperature. Thereafter, all cells were blocked with 5 % FBS and Triton X-100 and incubated with the primary antibodies described in Additional file 3: Table S1. The cells were then rinsed with PBS, and incubated with species-specific Alexa Fluor 488-, Alexa Fluor 555- or Alexa Fluor 647-conjugated secondary antibodies (1:500; Invitrogen, Massachusetts, USA); this was followed by Hoechst 33258 (0.5 μg/mL; Sigma-Aldrich, Missouri, USA) to counterstain the nuclei. The images were obtained using a universal fluorescence microscope (Axioplan2; Carl Zeiss AG, Oberkochen, Germany) or a confocal laser scanning microscope (LSM-710; Carl Zeiss AG, Oberkochen, Germany).

### CGH array analysis

Genomic DNA was extracted using a DNeasy Blood & Tissue Kit (QIAGEN, Hilden, Germany) according to the manufacturer’s protocol. Five hundred micrograms of genomic DNA was subjected to the Human CGH array (4X180K; Agilent Technologies, California, USA) according to the manufacturer’s protocol. All combinations (original cells and iPSCs) were analyzed. The data were analyzed using the Agilent Genomic Workbench. Rearrangements involving immunoglobulin gene regions and T-cell receptor gene regions that are rearranged in T-cell and B-cell lineages, respectively, were not taken into consideration in the analysis of the structural variations.

### Whole genome sequence analysis

One microgram of genomic DNA was subjected to whole genome sequence analysis. Sequencing libraries were constructed using a TruSeq DNA PCR-free library preparation kit (Illumina, California, USA). The sequences were analyzed using HiSeq2500 (Illumina, California, USA) in the rapid mode (150 bp, paired end). After alignment to the reference genome (hg19) using BWA [36] and removal of multiply aligned reads and duplicate reads, basecalls were performed with SAMtools [37]. Somatic variants were detected as previously described [38, 39]. We confirmed that all of the candidate somatic mutations consisting of single nucleotide substitutions were predicted to result in changes in the amino acid sequence by direct nucleotide sequence analysis. The SNVs inside T-cell receptor regions and immunoglobulin regions were excluded. If necessary, whole genome amplification using REPLI-g (QIAGEN, Hilden, Germany) was applied to the direct nucleotide sequence analysis.

### Microarray analysis

Total RNA was extracted with an RNAeasy Kit (QIAGEN, Hilden, Germany) and the RNA quality was assessed using an Agilent 2100 Bioanalyzer (Agilent Technologies, California, USA). Total RNA (100 ng) was reverse-transcribed, labeled with biotin using a 3’IVT Express Kit (Affymetrix, California, USA) and hybridized to a GeneChip® Human Genome U133 plus 2.0 Array (Affymetrix, California, USA). The arrays were washed and stained using a GeneChip Fluidics Station 450 (Affymetrix, California, USA) and then scanned with the GeneChip Scanner 3000 7G System (Affymetrix, California, USA) according to the manufacturer’s instructions. The raw probe intensity files were MAS5-normalized and log (base2) transformed by using GeneSpring GX 13.1 software (Agilent Technologies, California, USA). The gene set was filtered based on the expression levels to remove the genes that were not expressed in all samples. Principal component analysis (PCA) was performed using the normalized data. For the hierarchical clustering, the normalized data were calculated on the basis of Euclidean correlations with average linkages.

We used NCBI GEO microarray data GSE76832 for T-cell, DF and iPSCs established from them (T-cell(KA), DF(KA), TKA4, TKA9, eKA3 and KA11) [6].

### Reverse-transcription PCR

Total RNA was isolated using an RNeasy Mini Kit (QIAGEN, Hilden, Germany) and 1 μg of RNA was used to generate cDNAs by using a reverse transcription (RT) system (Promega, Wisconsin, USA). RT-PCR was performed as previously described [40]. Values were normalized to *ACTB*. Quantitative RT-PCR (qPCR) was performed on an ABI PRISM Sequence Detection System 7900HT (Applied BioSystems, Massachusetts, USA) by using SYBR premix ExTaq Tli RNaseH Plus (Takara Bio, Shiga, Japan). The primers are described in Additional file 4: Table S2.

### PCR amplification of genomic DNA

Genomic DNA was purified from HDFs, LCLs and hiPSCs using a DNeasy Kit (QIAGEN, Hilden, Germany). The PCR conditions were described previously (Additional file 4: Table S2) [41].

### Electrophysiological analysis

For the electrophysiology experiments, the culture medium was replaced with a physiological solution (118 mM NaCl, 2.5 mM KCl, 26 mM NaHCO_3_, 1 mM NaH_2_PO_4_, 10 mM glucose, 4 mM MgCl_2_, and 4 mM CaCl_2_). Tetrodotoxin (TTX, 1 μM) was bath-applied. The electrodes (5–8 MΩ) were filled with whole-cell pipette solution (120 mM potassium acetate, 20 mM KCl, 0.1 mM CaCl_2_, 5 mM MgCl_2_, 0.2 mM EGTA, 5 mM ATP, and 10 mM HEPES, pH 7.3) [42, 43]. The whole-cell recordings of GFP-expressing neurons were configured using an EPC-7 amplifier (HEKA Elektronik Dr. Schulze GmbH, Lambrecht/Pfalz, Germany) and a Digidata 1200 acquisition board (Axon Instruments, California, USA). The membrane potential was clamped at −60 mV. Membrane resistance (*R*m), series resistance (*R*s) and membrane capacitance (*C*m) were monitored. Only recordings with *R*m > 100 MΩ and *R*s < 20 MΩ were included in the analysis.

### Carbonyl cyanide m-chlorophenylhydrazone treatment

Neurons were cultured with 30 μM carbonyl cyanide m-chlorophenyl hydrazone (CCCP; Sigma-Aldrich, Missouri, USA) or DMSO for 48 h. The cells were then fixed, stained for βIII-TUBULIN and Complex-III Core I (CIII-Core I), and counterstained with Hoechst. The cytoplasmic area was extracted to quantify the IMM area of neurons, as shown in Fig. 5e. The IMM area of the neurons was quantified from the digitized values using IN CELL Analyzer 6000 (GE Healthcare, Chicago, USA).

### Oxidative stress analysis

The ROS levels were determined by measuring the CellROX fluorescence using the CellROX® Green Reagent to detect oxidative stress (Life Technologies, Massachusetts, USA). Briefly, the neurons were incubated with the CellROX Reagent for 30 min at 37 °C, after which they were washed with PBS and then fixed with 4 % PFA for 30 min at room temperature. Then, the cells were incubated with the primary antibody against βIII-TUBULIN (1:1000; Sigma-Aldrich, Missouri, USA) overnight at 4 °C, washed with PBS and incubated with an Alexa Fluor 555-conjugated secondary antibody (1:500; Invitrogen, Massachusetts, USA) for 1 h at room temperature. The fluorescence of the βIII-TUBULIN-positive neurons was measured by using an IN Cell Analyzer 6000 (GE Healthcare, Chicago, USA).

## Additional files

Additional file 1: Figure S1. In vitro differentiation analysis and karyotyping of LiPSC. (A) Representative karyotype of the established iPSC line from LCL-PB. (B) Quantitative analysis of immunocytochemistry for markers of three-germ layers; βIII-TUBULIN (ectoderm), αSMA (mesoderm) and AFP (endoderm) based on their fluorescence intensities (*n* = 3 independent experiments; means ± SEM; n.s., not significant; ANOVA). (C) Representative images of immunocytochemistry for the in vitro three-germ layer markers (βIII-TUBULIN, αSMA and AFP). Scale bars = 50 μm. (PDF 500 kb) Additional file 2: Figure S2. Somatic mutations in TiPSCs caused by the reprogramming process. **a** A substantial number of nonsynonymous variants were suggested to disappear in TKA4, all of which are located on chromosome 9, and Sanger sequencing showed an apparent mosaic loss of heterozygosity. An example of the Sanger sequence analysis of apparently lost nonsynonymous variant is shown. A small peak indicating a variant was observed in TKA4, suggesting a mosaic loss of heterozygosity. **b** The CGH analysis of TKA4 compared with the T-cells showed there were no copy number alterations in chromosome 9. However, a detailed inspection of the ratios of variants/reference bases revealed that the ratios were significantly different from 0.5 in the long arm of chromosome 9 of TKA4, supporting mosaic loss of heterozygosity in the region. Because the CGH analysis did not support copy number alterations (upper panel), these findings are more likely to represent somatic parental disomy involving the long arm of chromosome 9. A similar observation has recently been described [39]. (PDF 580 kb) Additional file 3: Table S1. List of antibodies. (PDF 400 kb) Additional file 4: Table S2. List of primers. (PDF 397 kb)

## Abbreviations

CGH: Comparative genomic hybridization
DF: Dermal fibroblasts
DF-iPSC: DF-derived iPSC
dNS method: Direct neurosphere conversion method
EBV: Epstein-Barr virus
iPSC: Induced pluripotent stem cells
LCL: Lymphoblastoid B-cell lines
LiPSC: LCL-derived iPSC
mDAN: Midbrain dopaminergic neuron
NS: Neurosphere
ROS: Reactive oxygen species
SNV: Single nucleotide variation
SV: Structural variation
TH: Tyrosine hydroxylase
TiPSC: T-cell derived iPSC
WGS: Whole genome sequencing
