# Supplementary figures and images for: Modeling neurological diseases with induced pluripotent cells reprogrammed from immortalized lymphoblastoid cell lines
Source: Mol Brain. 2016 Oct 3;9:88. doi: 10.1186/s13041-016-0267-6 (PMC5046991; doi:10.1186/s13041-016-0267-6)

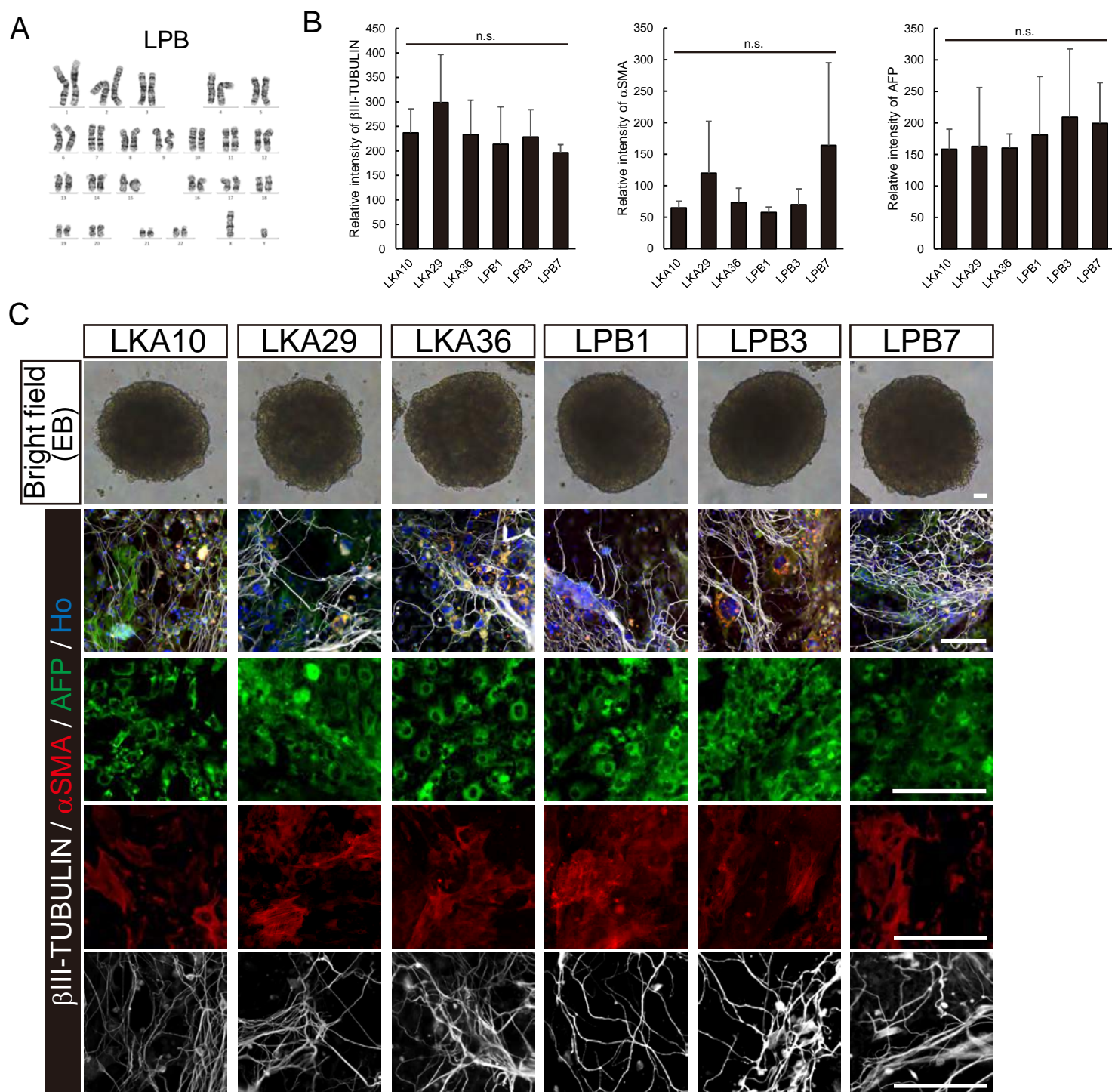

**Fig. S1**

Supplement: Additional file 1: Figure S1. — In vitro differentiation analysis and karyotyping of LiPSC. (A) Representative karyotype of the established iPSC line from LCL-PB. (B) Quantitative analysis of immunocytochemistry for markers of three-germ layers; βIII-TUBULIN (ectoderm), αSMA (mesoderm) and AFP (endoderm) based on their fluorescence intensities (n = 3 independent experiments; means ± SEM; n.s., not significant; ANOVA). (C) Representative images of immunocytochemistry for the in vitro three-germ layer markers (βIII-TUBULIN, αSMA and AFP). Scale bars = 50 μm. (PDF 500 kb) [file 13041_2016_267_MOESM1_ESM.pdf]

A

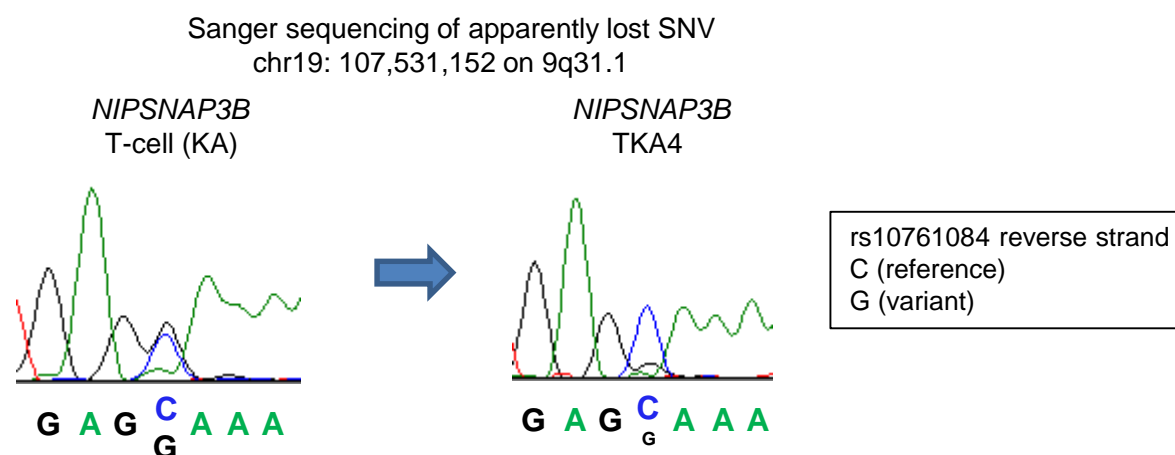

B

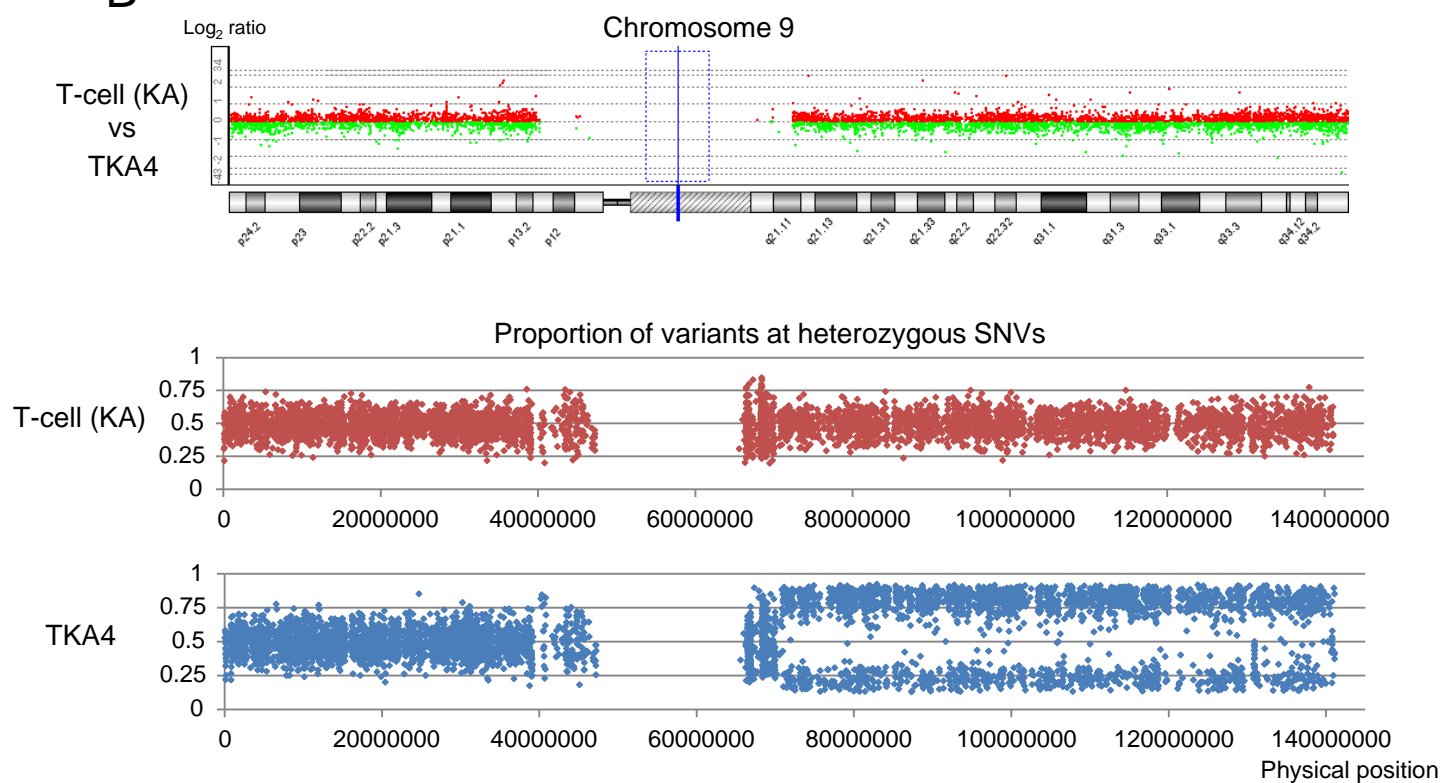

Fig. S2

Supplement: Additional file 2: Figure S2. — Somatic mutations in TiPSCs caused by the reprogramming process. a A substantial number of nonsynonymous variants were suggested to disappear in TKA4, all of which are located on chromosome 9, and Sanger sequencing showed an apparent mosaic loss of heterozygosity. An example of the Sanger sequence analysis of apparently lost nonsynonymous variant is shown. A small peak indicating a variant was observed in TKA4, suggesting a mosaic loss of heterozygosity. b The CGH analysis of TKA4 compared with the T-cells showed there were no copy number alterations in chromosome 9. However, a detailed inspection of the ratios of variants/reference bases revealed that the ratios were significantly different from 0.5 in the long arm of chromosome 9 of TKA4, supporting mosaic loss of heterozygosity in the region. Because the CGH analysis did not support copy number alterations (upper panel), these findings are more likely to represent somatic parental disomy involving the long arm of chromosome 9. A similar observation has recently been described [39]. (PDF 580 kb) [file 13041_2016_267_MOESM2_ESM.pdf]
