# Supplementary material for: Modeling neurological diseases with induced pluripotent cells reprogrammed from immortalized lymphoblastoid cell lines
Source: Mol Brain. 2016 Oct 3;9:88. doi: 10.1186/s13041-016-0267-6 (PMC5046991; doi:10.1186/s13041-016-0267-6)
Supplement: Additional file 3: Table S1. — List of antibodies. (PDF 400 kb) [file 13041_2016_267_MOESM3_ESM.pdf]

Table S1. List of antibodies

Antibody	Dilution	Source	Location
OCT4	1:200	Santa Cruz Biotechnology	Dallas, TX
TRA-1-60	1:1000	Millipore	Billerica, MA
βIII-tubulin	1:2000	Sigma-Aldrich	St Louis, MI
VGLUT1	1:4000	Synaptic Systems	Germany
GFAP	1:4000	DAKO	Glostrup, DK
GABA	1:2000	Sigma-Aldrich	St Louis, MI
TH	1:500	Millipore	Billerica, MA
Complex III-core I	1:200	Thermo Fisher Scientific	Waltham, MA

VGLUT1, vesicular glutamate transporter 1; GFAP, glial fibrillary acidic protein; GABA, gamma-aminobutyric acid; TH, tyrosine hydroxylase
