# Supplementary material for: Modeling neurological diseases with induced pluripotent cells reprogrammed from immortalized lymphoblastoid cell lines
Source: Mol Brain. 2016 Oct 3;9:88. doi: 10.1186/s13041-016-0267-6 (PMC5046991; doi:10.1186/s13041-016-0267-6)
Supplement: Additional file 4: Table S2. — List of primers. (PDF 397 kb) [file 13041_2016_267_MOESM4_ESM.pdf]

Table S2. List of primers

Name	Sequence
	**For genomic PCR**
Ex3 forward	ACATGTCACTTTTGCTTCCCT
Ex3 reverse	AGGCCATGCTCCATGCAGACTGC
Ex4 forward	AGGTAGATCAATCTACAACAGCT
Ex4 reverse	CTGGGTCAAGGTGAGCGTTGCCTGC
Ex6 forward	AGAGATTGTTTACTGTGGAAACA
Ex6 reverse	GAGTGATGCTATTTTTAGATCCT
Ex7 forward	GAGCCCCGTCCTGGTTTTCC
Ex7 reverse	CCACACAAGGCAGGGAGTAGCCAA
EBNA1 forward	ATCAGGGCCAAGACATAGAGA
EBNA1 reverse	GCCAATGCAACTTGGACGTT
EBNA2 forward	CATAGAAGAAGAAGAGGATGAAGA
EBNA2 reverse	GTAGGGATTCGAGGGAATTACTGA
LMP1 forward	ATGGAACACGACCTTGAGA
LMP1 reverse	TGAGCAGGATGAGGTCTAGG
BZLF1 forward	CACCTCAACCTGGAGACAAT
BZLF1 reverse	TGAAGCAGGCGTGGTTTCAA
OriP forward	TCGGGGGTGTTAGAGACAAC
OriP reverse	TTCCACGAGGGTAGTGAACC
GAPDH forward	ACCACAGTCCATGCCATCAC
GAPDH reverse	TCCACCACCCTGTTGCTGTA
	**For qPCR**
OCT4 forward	TTGGGCTCGAGAAGGATGTGGT
OCT4 reverse	TGCATAGTCGCTGCTTGATCGC
NANOG forward	TGAACCTCAGCTACAAACAG
NANOG reverse	TGGTGGTAGGAAGAGTAAAG
PAX6 forward	ACCACACCGGTTTCCTCCTTCACA
PAX6 reverse	TTGCCATGGTGAAGCTGGGCAT
NESTIN forward	TTCCCTCAGCTTTCAGGACCCCAA
NESTIN reverse	AAGGCTGGCACAGGTGTCTCAA
ACTB forward	TGAAGTGTGACGTGGACATC
ACTB reverse	GGAGGAGCAATGATCTTGAT
